# A New Preparation Method of Cement with Photocatalytic Activity

**DOI:** 10.3390/ma13235540

**Published:** 2020-12-04

**Authors:** Magdalena Janus, Szymon Mądraszewski, Kamila Zając, Ewelina Kusiak-Nejman

**Affiliations:** 1 Faculty of Civil and Environmental Engineering, West Pomeranian University of Technology in Szczecin, al. Piastów 50, 70-311 Szczecin, Poland; kamila.zajac@zut.edu.pl; 2 Building Materials and Construction Chemistry, Technische Universität Berlin, Gustav-Meyer-Allee 25, 13355 Berlin, Germany; szymon.madraszewski@tu-berlin.de; 3 Faculty of Chemical Technology and Engineering, West Pomeranian University of Technology in Szczecin, ul. Pułaskiego 10, 70-310 Szczecin, Poland; ekusiak@zut.edu.pl

**Keywords:** photoactive cement, titanium white, mechanical properties, NOx

## Abstract

The studies of some mechanical properties and photocatalytic activity of new cements with photocatalytic activity are presented. The new building materials were obtained by addition of semi-product from titanium white production. Semi-product was calcined at 300 and 600 °C for one, three, and five hours and then this material was added to cement matrix in an amount of 1 and 3 wt.%. New materials were characterized by measuring the flexural and compressive strength and the initial and the final setting time. The photocatalytic activity was tested during NOx photooxidation. The cement with photocatalytic activity was also characterized by sulphur content measurements. The measurement of reflectance percentage of TiO_2_-loaded cements in comparison with pristine cement and TiO_2_ photocatalyst calcined at 600 °C were also performed. It should be emphasized that although in some cases, the addition of photocatalyst reduced the flexural and the compressive strength of the modified cements, these values were still within the norm PN-EN 197-1:2012. It was also found that the initial and the final setting time is connected with the crystal size of anatase, and the presence of larger crystals significantly delays of the setting time. This was probably caused by a water adsorption on the surface of anatase crystals.

## 1. Introduction

Recently, there has been a lot of interest in the use of photocatalysts in building materials. The addition of a nanosized photocatalyst into the building elements allowed inducing a specific functionality such as a photocatalytic, a superhydrophilic, and some antimicrobial properties. The combination of a sunlight utilization with the functionally engineered materials has contributed to the aesthetic durability of the building elements and reducing environmental pollution [1,2]. The photoinduced superhydrophilicity of the photocatalytic materials allows spreading out some water droplets, generating a thin film of water, which eliminates dust from the surfaces and limits the optical interferences on the photocatalytic glass windows [3]. The disinfecting activity of the photocatalytic materials consists of damaging the cellular membranes of the bacteria. The antibacterial and an antifungal action of the photocatalysis was applied to control biological growth on the building surfaces [1,4]. The photocatalytic degradation of the pollutants contributed to the self-cleaning of the building surfaces as well as a removal of air pollutants from other industrial sectors [5]. 

Among various semiconductors, the most widely used photocatalyst is titanium dioxide. Within the numerous advantages of TiO_2_, its catalytic longevity, availability, and easy activation in ambient conditions as well as the compatibility with the conventional building materials should be mentioned [1,6]. The basic principle of the photocatalysis phenomenon is the activation of the photocatalyst particles by appropriate irradiation. The photocatalyst particles absorb a photon of the energy equal to or greater than a band gap energy (E_g_) of the semiconductor (for the base form of TiO_2_ it is attributed to UV irradiation). The electrons are promoted from a filled conduction band to an empty valance band, creating electron-hole pairs (e^−^–h^+^). Then, the sequential reactions lead to the formation of some reactive oxygen species (e.g., ·OH, HO_2_·, O_2_·^−^), which can effectively decompose pollutants to the simple inorganic compounds. In general, the high efficiency of the photocatalysis process is directly related to the following aspects: (1) the high absorption of irradiation by photocatalyst; (2) the high adsorption of pollutants molecules on the photocatalyst surface; (3) the quick charge separation; (4) the low charge recombination [7,8]. 

The example of NOx degradation can present the photocatalytic action of TiO_2_ towards an air pollution. In urban areas, nitrogen oxides are the most problematic pollutants affecting human health [9]. At the beginning of the photocatalytic process, the mechanism involves a generation of some oxidative species (reactions 1–3). The hydroxyl radicals ^·^OH and superoxide ions O_2_·^−^ react with the nitrogen oxides to form harmless nitrates through the reactions 4–7. Namely, the ·OH radical reacts with the adsorbed NO to form HNO_2_ (reaction 4). The formed HNO_2_ further reacts with the ^·^OH radical to create NO_2_ and water (reaction 5). Next, NO_2_ reacts with ·OH to generate HNO_3_ (reaction 6). Simultaneously, O_2_·^−^ reacts with the nitrogen oxides to form nitrates (reaction 7) [10,11]. It is worth pointing out that the intermediate NO_2_ is considerably more toxic than NO. Hence, it is favorable to attain the higher selectivity of NO to NO_3_^−^ than NO to NO_2_ [12].

TiO_2_ + hυ → TiO_2_* (h^+^ + e^−^) (1)

OH^−^ + h^+^ → ·OH (2)

O_2_ + e^−^ → O_2_·^−^ (3)

NO + ·OH → HNO_2_ (4)

HNO_2_ + ·OH → NO_2_ + H_2_O (5)

NO_2_ + ·OH → HNO_3_ (6)

NO + O_2_·^−^ → NO_3_^−^ (7)

The dominant materials in the construction industry have remained for years cement-based materials or composites. However, apart from their structural function, recently, the modern properties were added to by addition of steel fiber reinforcement, nano additives, or by the addition of self-compacting substances [13]. Photocatalytic cements were first prepared in Japan at the start of the 1990s [14]. Presently, there are conceived facades, streets, and the pavements from photocatalytic cements [15,16,17]. Thus far, TiO_2_ containing cement-based materials was prepared using different techniques, which still faces a serious restriction. 

Heterogeneous photocatalysis is a surface process. Therefore, nanomaterials are often immobilized on an appropriate building substrate. The photocatalytic coating for building materials were obtained by using dip-coating, spin coating, spraying, or brushing [6]. Feng et al. [18] prepared the photocatalytic TiO_2_/cement composite by a smear method through the floating emulsion of TiO_2_ onto the pre-wet surface of the cement mortar. They showed that TiO_2_ particles were dispersed equally on the surface of the cementitious material and the prepared mortar had a good cyclical photocatalytic performance. Baltes et al. [6] showed that the application of the dip-coating technique led to the high photocatalytic activity, but that low mechanical resistant layers were obtained. The methods using TiO_2_ surface treatment for the cement-based materials appeared to be problematic due to the weak adhesion between the photocatalytic coating and the building material. The poor weathering resistance is evidence for this, especially in some aggressive outdoor environments [19]. A valuable resource to prevent the release of TiO_2_ particles from the building surface is SiO_2_, owing to its pozzolanic activity with cement-based materials. Wang et al. [20] prepared SiO_2_/TiO_2_ composites with different deposited densities of TiO_2_ on SiO_2_ spheres, which were used for the surface coating of a cement-based material. The authors showed good photocatalytic activity and the durability of the SiO_2_/TiO_2_ composites. However, Mendoza et al. [21] indicated that despite SiO_2_ action as an interlayer between TiO_2_ and the substrate, it could not effectively stabilize TiO_2_ coating. 

Hernández-Rodríguez et al. [22] proposed a partial replacement of the cement with TiO_2_. The photocatalyst was only incorporated into a one-centimeter-thick surface layer on the cementitious specimens. A photocatalyst is also often embedded into the mass of cementitious material by simple intermixing of both substrates [23,24]. On the one hand, it leads to lower photocatalytic efficiency due to partial use of TiO_2_ particles, which are active only when situated to the surface [19]. On the other hand, the effect of TiO_2_ addition to cementitious mass leads not only to photocatalytic activity, but also improves the mechanical properties of cementitious materials. The enhancement of mechanical properties by filling the pores and interaction with the other components of cement were observed [6,22,25].

Despite the multiple examples of the photocatalytic cementitious materials, most of the products are not competitive enough and create difficulties during the production and applications in the real conditions [22]. There is, therefore, a need to discern new photocatalytic materials concerning new synthesis methods and new incorporation strategies into the building binders. 

This paper aims to present the photocatalytic cementitious materials preparation from cement and semi-product of TiO_2_. The proposed method may lower the price of the photoactive cement. The obtained materials were analyzed in detail towards photocatalytic and some mechanical properties. 

## 2. Materials and Methods 

### 2.1. Materials

Portland cement CEM I 42.5 N (Holcim, Germany) as base material was used. Standard sand, according to the standard EN 196-1 “Methods of testing cement. Determination of strength” was used in the mixture. The semi-product from titanium white production supplied by Grupa Azoty Zakłady Chemiczne ‘Police’ S.A. (Police, Poland) was used as a starting material. This material was downloaded from installation for titanium white production by sulfate method; the material was taken from drum filters before calcination. The semi-product was calcined at 300 and 600 °C for 1, 3, and 5 h. 

### 2.2. Specimens Preparation

The specimens 40 × 40 × 160 mm were produced according to the EN 196-1 standard with water to binder ratio w/b = 0.4 and cement to standard sand ratio 1:3. Cement was replaced by the photocatalyst in 1 and 3 wt.% of cement. For each type of mortars, 6 specimens were produced. Masses needed for the preparation of 2 types of specimens in Table 1 are presented.

The standard mixer with stainless steel bowl with a capacity of 5 l according to EN 196-1 was used. First, water was poured into a bowl and cement was added. The casting molds containing fresh samples were wrapped with stretch film and stored in room conditions for 24 h. All specimens were demolded after 1 day and were cured in tap water for the next 27 days.

### 2.3. The Compressive and the Flexural Strength Measurements

After 28 days, specimens for the flexural and the compressive strength were tested. The flexural and the compressive strength measurements in accordance with the EN 196-1 standard were carried out. A standard testing machine (i.e., ToniNORM 2010.040, Toni/Technik, Berlin, Germany) was used.

### 2.4. The Initial and the Final Setting Time (Vicat Needle Test)

Vicat Apparatus (ToniSET COMPACT version 05/00, Berlin, Germany) was used to determine the setting time of cement paste. For each mortar type, 2 specimens were prepared. The mortar preparation and the setting time measurements were run according to the EN 196-3 standard. During measurements, specimens were cooled with 20 °C water. Water to binder ratio was w/b = 0.3. The time when the needle stops 6 mm from the base plate was recorded as the time for the initial setting. The final setting was defined as the time when the needle only made a 0.5-mm mark on the surface. 

### 2.5. The Crystalline Structure, UV-Vis/DR Measurments, and Sulphur Content

The crystalline structure of the photocatalysts was characterized by X-ray powder diffraction (XRD) analysis (X’Pert PRO Philips diffractometer, Eindhoven, Netherlands) using CuK_α_ radiation. The mean size of crystallite was calculated from full-width at half-maxima (FWHM) of corresponding X-ray diffraction peaks using Scherrer’s formula, where λ is the wavelength of the X-ray radiation (λ = 1.54056 nm CuK_α_), β is the full-width at half maximum (rad) and is the reflect angle. The width of the peak at half maximum was calculated after correction of the instrument error. The presented method was applied to estimate of change in the crystallite size of TiO_2_ particles. The materials were characterized by the UV-VIS/DR (diffiuse-reflectance) technique using the Jasco V-530 (Tokyo, Japan) spectrophotometer equipped with the integrating sphere accessory for the diffuse reflectance spectra (BaSO_4_ was used as a reference). 

The content of sulphur (wt.%) in tested photocatalysts, as well as TiO_2_-modified cement were determined by means of CS230 elemental analyzer (Leco Co., St. Joseph, MI, USA). The BCS-CRM powder containing 1.48 wt.% of inorganic sulphur was used as a calibration standard.

### 2.6. NOx Decomposition

**Figure 1:**
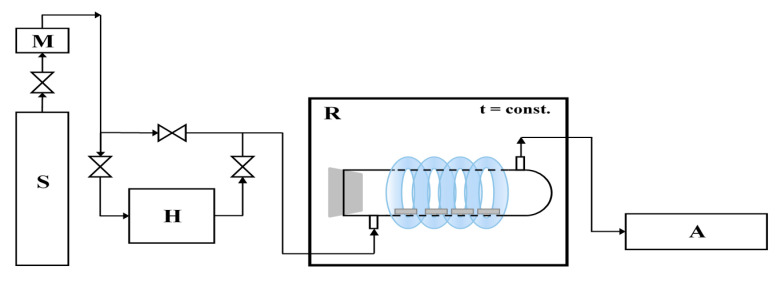
The scheme of installation to photocatalytic removal of NOx. S—a source of pollution; M—mass flower; H—humidifier; R—photocatalytic reactor with irradiation source; A—NOx analyzer.

In our previous work [26,27], the NO gas (1.989 ppm ± 0.040 ppm, Air Liquid) was used as model pollution in photocatalytic tests. NOx removal was evaluated using the experimental installation (Figure 1).

The studied cement plate (one at dimensions of 8 × 4 × 1 cm) was placed in the central part of the cylindrical reactor (Pyrex glass; Ø × H = 9 cm × 32 cm). The NO (II) was diluted with humidified synthetic air in ratio 1:1. The process was carried out continuously with a gas flow 500 cm^3^/min. At the beginning of the process, the dark conditions were maintained until NO concentration reached equilibrium (about 1 ppm during about 35 min). Then the 4 × 22 W UV lamps (Philips) were turned on for 30 min. The irradiation sources surrounded the rector and were characterized by the cumulative intensity of 100 W/m^2^ UV and 4 W/m^2^ Vis. The temperature of the whole system was stable at the level of 22 °C by using the thermostatic chamber. The NO and NO_2_ concentrations were continuously measured in the outlet of the reactor using chemiluminescent NOx analyzer (T200, Teledyne).

## 3. Results and Discussion

### 3.1. The Compressive and the Flexural Strength

**Figure 2:**
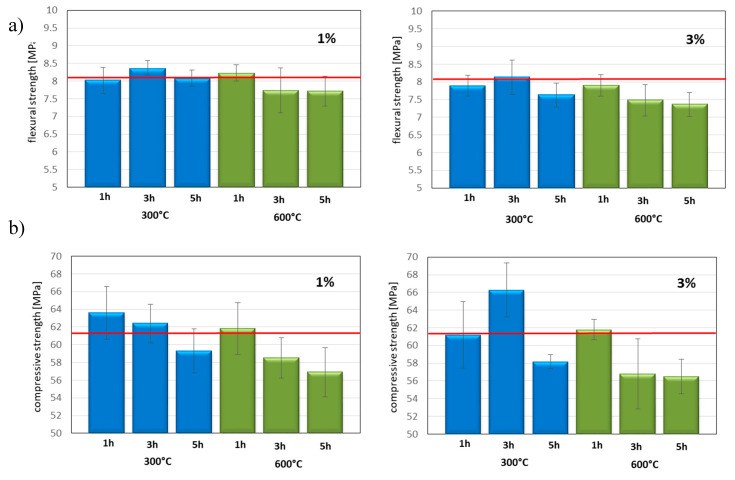
(**a**) The flexural and (**b**) the compressive strength of CEM I 42.5 N with the addition of 1; 3 of photocatalyst TiO_2_. In the red line, the compressive strength (61.5 MPa) and flexural strength (8.1 MPa) of pure CEM I 42.5 is presented.

**Figure 3:**
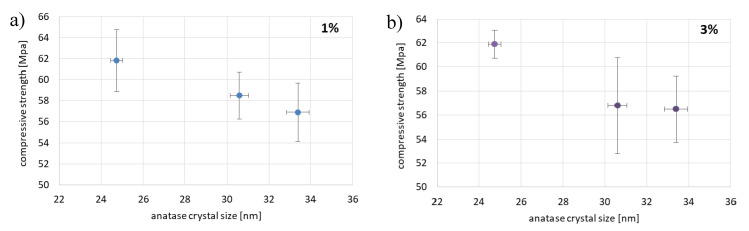
The relationship between the size of anatase crystals of titanium dioxde and the compressive strength for cement with (**a**) 1 wt.% and (**b**) 3 wt.% addition of TiO_2_ calcined at 600 °C for 1, 3, and 5 h. Standard deviations were indicated.

The compressive and the flexural strength of unmodified cement and cement with the addition of 1 and 3 wt.% of TiO_2_ modified at 300 and 600 °C specimens were measured. The obtained results in Figure 2a,b are presented. The red line in all graphs represents the value of unmodified cement. The graphs also show standard deviations for the mean measurement values. As it can be seen in Figure 2a, the value of the compressive strength of unmodified cement amounted to 61.5 MPa. The highest value of the compressive strength was observed for specimens with 3 wt.% of TiO_2_ calcined at 300 °C for 3 h, and amounted to 66.3 MPa. The lowest value of the compressive strength was found for the specimens with the addition of 3 wt.% TiO_2_ calcined at 600 °C for 5 h, and amounted to 56.5 MPa. As it can be seen in Figure 2b, the value of the flexural strength of unmodified cement amounted to 8.1 MPa. The addition of 1 and 3 wt.% of TiO_2_ decreased the flexural strength of specimens in almost all cases. The highest value of flexural strength was observed for a specimen with 1 wt.% of TiO_2_ calcined at 300 °C for 3 h, and amounted to 8.37 MPa. The lowest value of the flexural strength was obtained using specimen with the addition of 3 wt.% of TiO_2_ calcined at 300 °C for 3 h, and this value amounted to 7.36 MPa. It seems that the mechanical properties (the compressive and the flexural strength) of cements depended on the crystal size of anatase form of used titanium dioxide (Figure 3). The crystal size of anatase obtained at 300 °C amounted to about 8 nm, and it can be seen that with the addition of this material the values of the compressive and the flexural strength was close to the value of these parameters for unmodified cement. When the crystal size of anatase increased, the compressive and the flexural strength decreased. 

This behavior was especially visible in the case of addition of 3 wt.% of TiO_2_ modified at 600 °C. While the anatase crystallite size increased with the increasing of calcination time, the decrease in the compressive and the flexural strength was observed. In Figure 3, the relationship between the size of anatase crystals of titanium dioxde and the compressive strength for cement with 1 and 3 wt.% of TiO_2_ calcined at 600 °C for 1, 3, and 5 h is presented.

It should be emphasized that although in some cases, the addition of photocatalyst reduced the flexural and the compressive strength of the modified cements, these values were still within the norm PN-EN 197-1:2012.

### 3.2. The Initial and the Final Setting Time

The values of the initial and final setting time of tested specimens in Table 2 are presented. As it can be seen, in the case of 1 wt.%, addition of TiO_2_ slightly extended the initial setting time in comparison to unmodified cement. In the case of TiO_2_ modified at 300 °C regardless of the modification time, the initial setting was medium 30 min later, and the final setting time was around 50 min later than the initial setting time of unmodified cement. The initial setting time for TiO_2_ modified at 600 °C was medium 40 min later, and the final setting time was around 70 min later than the initial setting time of unmodified cement. In the case of adding 1 wt.% of the photocatalyst, we did not observe any relationship between the initial and final setting time and the calcination time of TiO_2_ at temperatures of 300 and 600 °C.

A different situation was observed when the photocatalyst was added in an amount of 3 wt.%. When this amount of photocatalyst was added to cement, it was possible to see the influence of calcination temperature of TiO_2_ on the initial and the final setting time. For TiO_2_ modified at 600 °C, it was impossible to give the medium value of initial and the final setting time because the calcination time influences the setting time. When the calcination time of photocatalyst increased from 1 to 5 h, the initial setting time also increased from 232 to 255 min and the final setting time increased from 315 to 366 min. Our earlier studies about the addition of nitrogen-modified TiO_2_ to the CEM I 42.5 N [28] and studies presented by Hernández-Rodríguez et al. [22] about the addition of commercial TiO_2_ P25 to CEM I 52.5 R showed that photocatalysts acted as a setting accelerator. In these studies, there was an opposite situation, and these photocatalysts were the setting retarder.

One of the reasons for delaying the setting time could be the presence of sulfate species on the TiO_2_ surface. The sulfate groups were present because this material was taken from the technological line of titanium white production by sulfate method. There are many publications describing the influence of sulfur on cement. Gies at al. [29] found that upon the absence of alkalis, increasing sulfate contents in belite-rich cement clinkers induced a significantly higher belite content, which is associated with a decrease in the alite content and, consequently, a reduction in the compressive strengths after 2 days of the resulting cements. The influence of SO_3_ in clinker on the properties of cement might depend on the C_3_A content of the cement because SO_3_ can react with C_3_A in the pore solution in cement in an early stage of ageing. However, few studies have focused on the heat of hydration and drying shrinkage of Portland cement with high-SO_3_ clinker with different C_3_A contents and added gypsum [30].

To confirm the effect of sulfur on the initial and the final setting time of modified cement, the measurements of sulfur in photocatalysts were done. The obtained results in Table 3 are presented. 

Because the amount of added photocatalysts to cement amounted to 1 and 3 wt.%, and in the photocatalyst, the amount of sulfur reached minimally from 0.67 to maximally 2.48 wt.%. The amount of 0.06wt% of sulphur additional introduced to cement was too small to influence the initial and the final setting time, and there was no dependence between the initial and the final setting time and the amount of sulfur in photocatalysts. The only visible relationship during setting time concerned cements modified by the addition of 3 wt.% of photocatalysts modified at 600 °C for 1, 3, and 5 h. 

**Figure 4:**
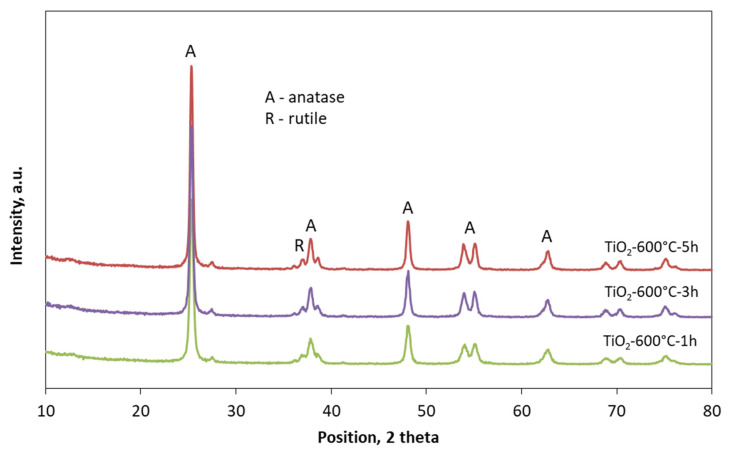
XRD patterns of TiO_2_ modified at 600°C for 1, 3, and 5 h.

The observed changes could be connected with the changes in crystallographic structure of TiO_2_. In Figure 4, the XRD patterns of TiO_2_ modified at 600 °C for 1, 3, and 5 h are presented.

**Figure 5:**
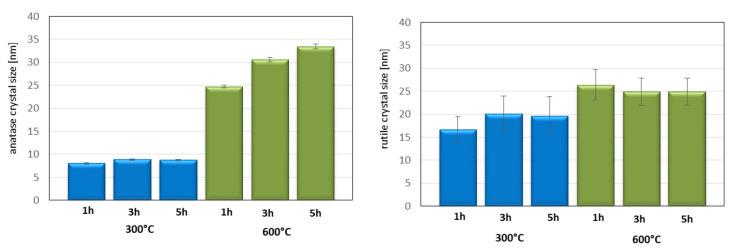
The crystal size of anatase and rutile presented in titanium white calcinated at 300 and 600 °C for 1, 3, and 5 h. Standard deviations were indicated.

In Figure 5, the calculated values of crystal size of anatase and rutile crystals of TiO_2_ modified at 300 and 600 °C for 1, 3, and 5 h is presented. As can be seen, the modification temperature influenced the crystal size of anatase. TiO_2_ modified at 300 °C had a smaller crystal size (around 8 nm) than TiO_2_ modified at 600 °C. In the case of TiO_2_ modified at 600 °C, it was even possible to see that the modification time influenced the crystal size of anatase. After 1 h of TiO_2_ calcination at 600 °C, the crystal size amounted to 24 nm, and after 5 h of calcination, the crystal size increased to 33 nm. The initial and the final setting time was connected with a crystal size of anatase, and the presence of larger crystals successfully delay the setting time. This probably caused by water adsorption on the anatase surface. Water adsorbed on the anatase surface was not available immediately for cement particles, and there was a reason for the delay of setting time.

### 3.3. NOx Decomposition

**Figure 6:**
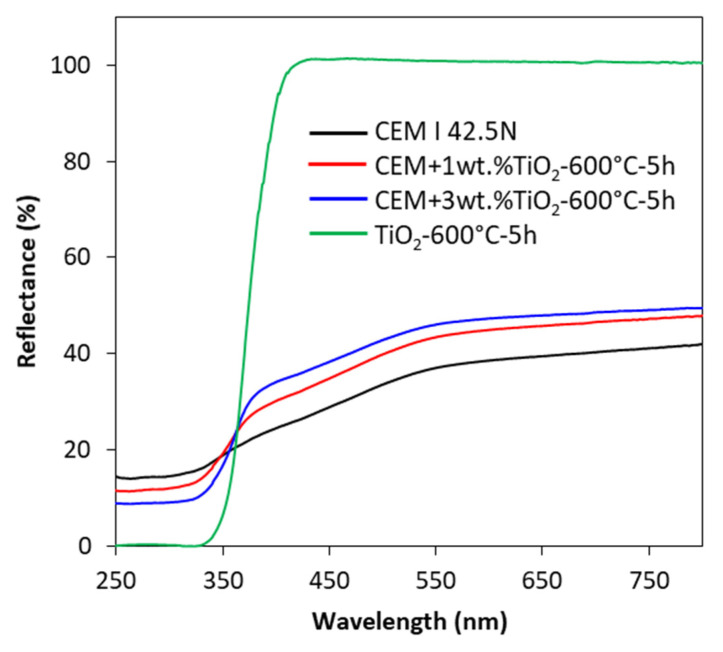
The UV-Vis/DR spectra of pure cement and photocatalyst calcinated at 600 °C for 5 h and cement with 1 and 3 wt.% addition of photocatalyst calcinated at 600 °C for 5 h.

The absorption abilities, determined on the basis of the measurement of reflectance percentage, of TiO_2_-loaded cements in comparison with pristine cement and TiO_2_ photocatalyst calcined at 600 °C are presented in Figure 6. The character of the UV-Vis/DR spectra of modified cements was similar to the spectra of unmodified cement. The spectra of the photocatalyst were found to be typical for white TiO_2_-based nanopowders. Despite the small quantities of TiO_2_ photocatalyst added (1 and 3 wt.%), a characteristic band could be found, as in the case with a pristine photocatalyst. What is more, the color of the modified cement plates was lighter due to the addition of white TiO_2_, and the absorption of the radiation in the range of UV increased with the increase of the amount of added photocatalyst. The opposite observation was found for visible region.

**Figure 7:**
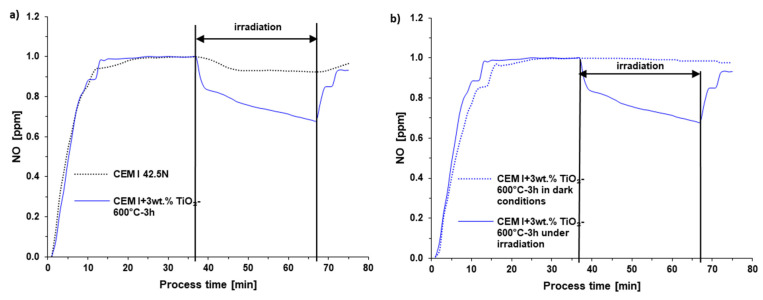
(**a**) The comparison of pure and modified cement under irradiation and (**b**) the comparison of influence of dark and irradiation conditions during process of NOx decomposition on selected cement samples.

In Figure 7, the comparison of pure and modified cement under irradiation and the comparison of influence of dark and irradiation conditions during process of NOx decomposition on selected cement samples was presented. In Table 4 the photocatalytic activity of unmodified and modified cements is presented. The activity of obtained materials during NOx removal were tested. The reference sample, unmodified cement CEM I showed the removal of NOx on the level of about 6.3%. 

The same observation in relation to the blank sample was presented by Xu et al. [31]. They found that using reference cement composites without any TiO_2_, the NOx concentration slowly decreased by 6% during 15 min of irradiation. It is worth pointing out that in our studies, the photolysis of tested gas amounted to 1.3% under the same conditions and the same irradiation source. The application of TiO_2_ in cement mortars involved the degradation of NOx on the photocatalytic path, which can be observed as the unambiguous decrease of NOx concentration directly after turning on the irradiation. The increasing temperature of calcination of TiO_2_ loaded to the cement matrix caused the increase of the NOx degradation rate. A 3 wt.% addition of photocatalyst calcined at 300 °C for 3 h to cement caused that this material removed 10.9% of NO, while 3 wt.% addition of photocatalyst calcined at 600 °C for 3 h to cement caused this material to remove 25.3% of NO. There was a typical behavior with a higher amount of TiO_2_ in the cement matrix to cause a higher amount of NO(II) removal. For example, when using 1 wt.% of TiO_2_ calcined at 300 °C for 1 h as an additive to cement, 10.4% of NO was successfully oxidized, while utilizing 3 wt.% of the same photocatalyst caused 17.4% of NO removal. 

## 4. Conclusions

The semi-product from the installation of titanium white production by sulfate method can be used after calcination as an additive to cement mortars to give the photocatalytic properties to these materials. All photocatalytic samples degraded NOx during irradiation time, achieving higher NOx removal rate with higher TiO_2_ dosage in cement materials. Addition of the cement mortar sometimes slightly decreased and sometimes slightly increased the mechanical properties, but these values were still within the norm PN-EN 197-1:2012. Used semi-product after calcination at 300 and 600 °C included from 0.67 to 2.48 wt.% of sulphur, but this amount did not have an influence on initial and final setting time of obtained mortars. Obtained materials have photocatalytic activity, their activity was tested during NO (II) decomposition. Cement with 3 wt.% addition of TiO_2_ calcined at 300 °C for five hours decomposed 17.4% of NO(II) under UV light irradiation. 

## Figures and Tables

**Table 1:** Mass of materials used for the production of mortar specimens.

Materials	Mass of Used Materials [g]	
	1%	3%
CEM I 42.5 N	444.5	436.5
TiO_2_^1^	4.5	13.5
Standard Sand	1350	1350
water	180	180

^1^ TiO_2_ calcined at 300 and 600 °C for 1, 3, and 5 h.

**Table 2:** The values of initial and final setting time of CEM I and CEM I with the addition of 1; 3 wt.% of TiO_2_ photocatalysts.

Samples	The Initial Setting Time [min]	The Final Setting Time [min]
CEM I 42.5 N	218	305
CEM + 1 wt.%TiO_2_-300 °C-1 h	244	354
CEM + 1 wt.%TiO_2_-300 °C-3 h	259	375
CEM + 1 wt.%TiO_2_-300 °C-5 h	244	332
CEM + 3 wt.%TiO_2_-300 °C-1 h	217	296
CEM + 3 wt.%TiO_2_-300 °C-3 h	220	292
CEM + 3 wt.%TiO_2_-300 °C-5 h	203	289
CEM + 1 wt.%TiO_2_-600 °C-1 h	246	379
CEM + 1 wt.%TiO_2_-600 °C-3 h	273	375
CEM + 1 wt.%TiO_2_-600 °C-5 h	263	384
CEM + 3 wt.%TiO_2_-600 °C-1 h	232	315
CEM + 3 wt.%TiO_2_-600 °C-3 h	245	344
CEM + 3 wt.%TiO_2_-600 °C-5 h	255	366

**Table 3:** The mass% of sulphur in photocatalysts.

Samples	Mass% of Sulfur
TiO_2_	2.48
TiO_2_-300 °C-1 h	2.33
TiO_2_-300 °C-3 h	2.30
TiO_2_-300 °C-5 h	2.32
TiO_2_-600 °C-1 h	1.09
TiO_2_-600 °C-3 h	0.76
TiO_2_-600 °C-5 h	0.67

**Table 4:** NO (II) removal on cement plates with the addition of 1 and 3 wt. % of TiO_2_ calcined at 300 and 600 °C for 1, 3, and 5 h.

Samples	NO (II) Removal [%]
CEM I 42.5N	6.3
CEM + 1 wt.%TiO_2_-300 °C-1 h	6.2
CEM + 1 wt.%TiO_2_-300 °C-3 h	9.1
CEM + 1 wt.%TiO_2_-300 °C-5 h	10.4
CEM + 3 wt.%TiO_2_-300 °C-1 h	12.8
CEM + 3 wt.%TiO_2_-300 °C-3 h	10.9
CEM + 3 wt.%TiO_2_-300 °C-5 h	17.4
CEM + 1 wt.%TiO_2_-600 °C-1 h	10.3
CEM + 1 wt.%TiO_2_-600 °C-3 h	13.7
CEM + 1 wt.%TiO_2_-600 °C-5 h	15.6
CEM + 3 wt.%TiO_2_-600 °C-1 h	15.8
CEM + 3 wt.%TiO_2_-600 °C-3 h	25.3
CEM + 3 wt.%TiO_2_-600 °C-5 h	16.3
